# Clinical update on head and neck cancer: molecular biology and ongoing challenges

**DOI:** 10.1038/s41419-019-1769-9

**Published:** 2019-07-15

**Authors:** Elham Alsahafi, Katheryn Begg, Ivano Amelio, Nina Raulf, Philippe Lucarelli, Thomas Sauter, Mahvash Tavassoli

**Affiliations:** 1 Head and Neck Oncology Group, Centre for Host Microbiome Interaction, King’s College London, Hodgkin Building, London, SE1 1UL UK; 2 Medical Research Council, Toxicology Unit, Leicester University, Leicester, LE1 9HN UK; 3 Faculté des Sciences, de La Technologie et de La Communication, University of Luxembourg, 6, Avenue Du Swing, Belvaux, 4367 Luxembourg

**Keywords:** Molecular biology, Prognostic markers

## Abstract

Head and neck squamous cell carcinomas (HNSCCs) are an aggressive, genetically complex and difficult to treat group of cancers. In lieu of truly effective targeted therapies, surgery and radiotherapy represent the primary treatment options for most patients. But these treatments are associated with significant morbidity and a reduction in quality of life. Resistance to both radiotherapy and the only available targeted therapy, and subsequent relapse are common. Research has therefore focussed on identifying biomarkers to stratify patients into clinically meaningful groups and to develop more effective targeted therapies. However, as we are now discovering, the poor response to therapy and aggressive nature of HNSCCs is not only affected by the complex alterations in intracellular signalling pathways but is also heavily influenced by the behaviour of the extracellular microenvironment. The HNSCC tumour landscape is an environment permissive of these tumours’ aggressive nature, fostered by the actions of the immune system, the response to tumour hypoxia and the influence of the microbiome. Solving these challenges now rests on expanding our knowledge of these areas, in parallel with a greater understanding of the molecular biology of HNSCC subtypes. This update aims to build on our earlier 2014 review by bringing up to date our understanding of the molecular biology of HNSCCs and provide insights into areas of ongoing research and perspectives for the future.

## Facts

- The heterogeneous nature of HNSCC at the molecular level has hindered both the identification of specific targets and development of targeted therapeutics for this group of tumours.
- Advances in strategies in dissecting the features of the HNSCC genome, transcriptome and metabolome have revealed new altered targets. But this has not yet resulted in clinical improvements in the management of these cancers.
- Current treatment strategies are very toxic, highlighting the need for treatment stratification using validated biomarkers to improve treatment outcome and reduce toxicity and cost of HNSCC treatment.
- Radiotherapy resistance remains a major cause of HNSCC poor survival rates. Understanding the underlying molecular mechanism of RT resistance should significantly impact patient survival outcomes, but requires a multidisciplinary approach combining imaging and molecular profiling.
- EGFR inhibitors, the only approved targeted drugs, have limited efficacy with the mechanisms of inherent and acquired resistance remaining unresolved.
- There has been a significant increase in the incidence of HPV-positive HNSCC, a subgroup with more favourable prognosis. Strategies for treatment de-escalation to reduce toxicity are urgently required.
- New, tailorable treatments such as immunotherapy have become highly valuable in the treatment of HNSCC. However, the importance of both the tumour microenvironment and the role of tumour immunity in pathogenesis and treatment response needs further understanding.
- A possible relationship between the oral microbiome and HNSCC has been reported, warranting further research into the influence of the oral microbiome on subsequent development of HNSCC.

## Open questions

- How can HNSCC therapies, alone or in combination (radiotherapy, chemotherapy, targeted drugs and immunotherapy) be made more effective, to achieve a good prognosis while minimising undesirable treatment effects?
- For individuals with HPV-linked HNSCC, is it safe and effective to use less aggressive treatment than the usual highly toxic therapies?
- How can advances in precision medicine (and identification of biomarkers) help clinicians to personalise treatment and predict outcomes, based on the patient’s unique biochemistry and genetic profile?
- Can the relationship between tumour and microenvironment (including the input of the immune system and influence of the microbiome) help us either treat, stratify patients or prevent oncogenesis?
- To what extent is the HNSCC epigenome contributing to evolution of the tumour? Will the epigenome be the next-generation pharmacological target for HNSCC?

## Introduction

**Fig. 1:**
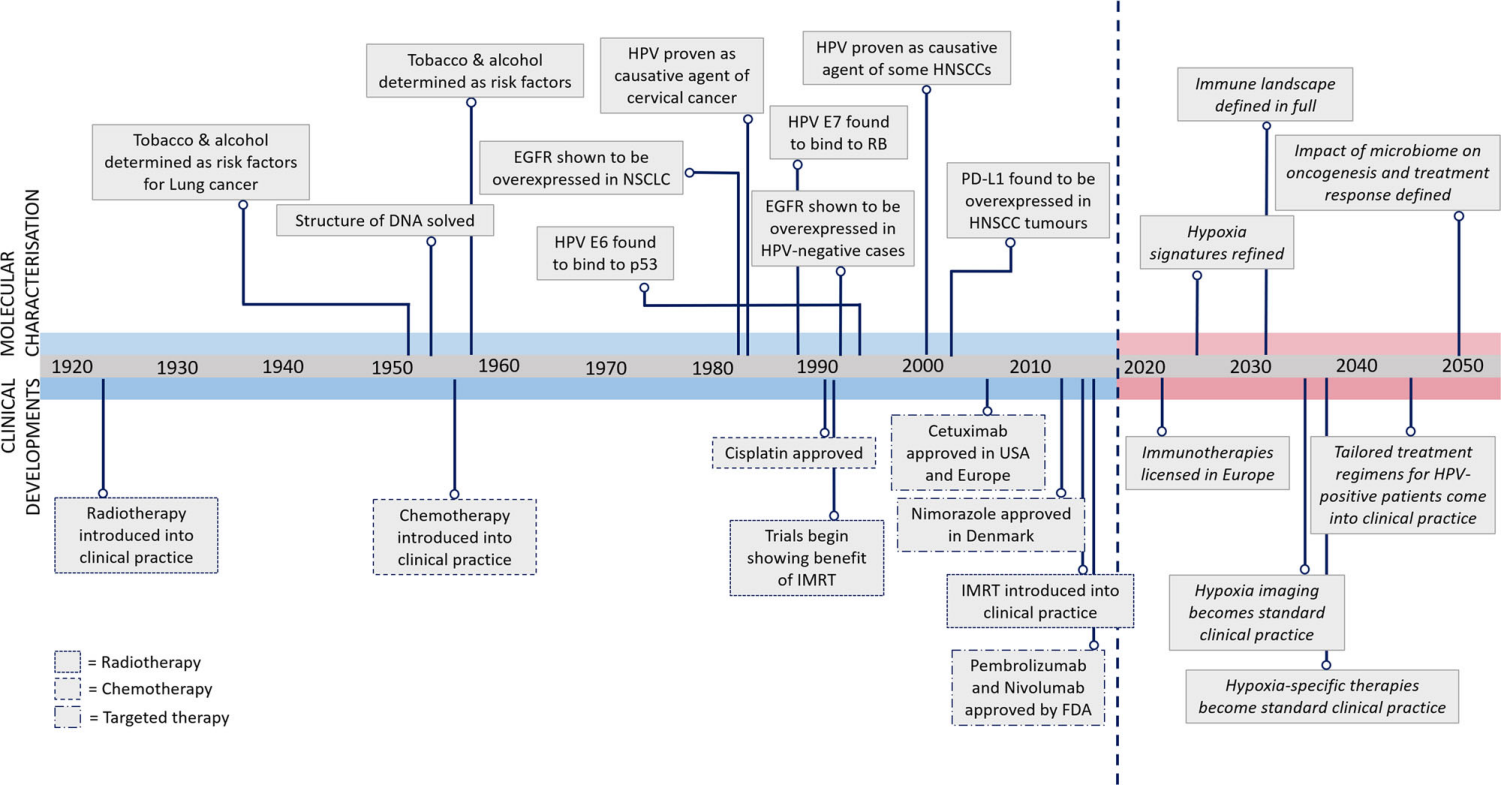
Timeline of the molecular characterisation and therapeutic innovations in head and neck cancers, and future perspectives. Interestingly, the major advances in our understanding of HNSCCs have only been made in the past 20–30 years. Also, some major discoveries concerning the molecular characterisation of HNSCCs have been made almost 20 years after similar discoveries in other cancers. For example, where HPV was proven unequivocally to cause cervical cancers around 1983, the same discovery was not made for HNSCCs until around the year 2000. It is not so hard to understand therefore why therapeutic options for HNSCCs are so far behind, when our understanding of the molecular biology of these diseases only began to develop over the past 2–3 decades. Dates are not exact, and the details presented are by no means exhaustive^10–22^

Head and neck squamous cell carcinomas (HNSCCs) are the sixth most common malignancy worldwide, accounting for over 500,000 new cases annually. Long-term tobacco use, consumption of alcohol and infection with high-risk types of Human Papilloma Virus (HPV) are considered the main oncogenic drivers^1,2^. Treatment involves surgical eradication, radiotherapy (RT) and chemotherapy (CT). All modalities severely reduce quality of life, and are largely ineffective. Most of the developments towards understanding this disease have occurred in the past few decades, but have fallen short of clinically meaningful discoveries (Fig. 1). Efforts to improve treatment efficacy have been largely without success, highlighting an urgent need for more effective therapies, alongside clinically relevant biomarkers to stratify patients and improve treatment outcomes.

**Fig. 2:**
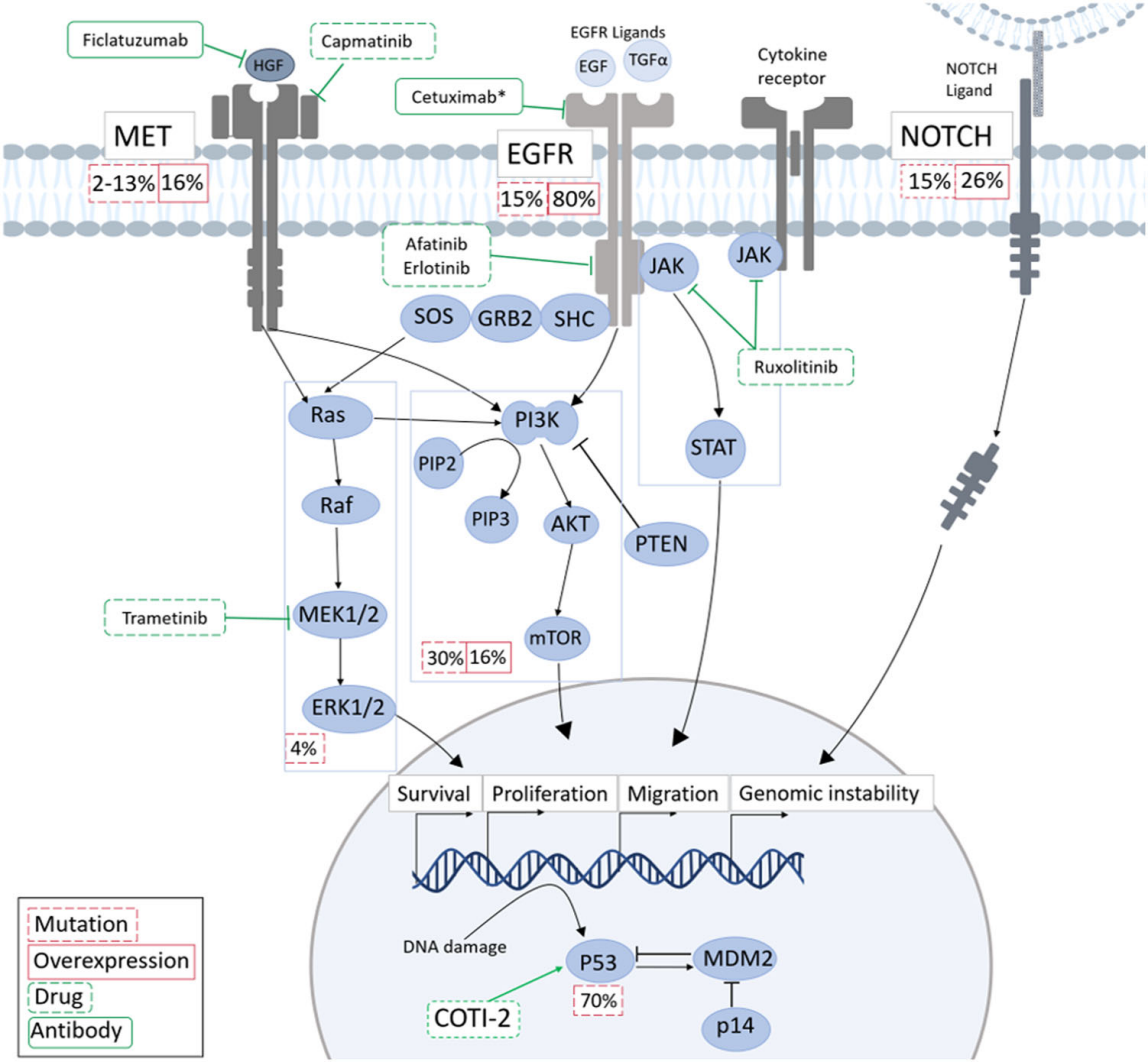
The genetic alterations in HPV-negative HNSCCs. EGFR, MET and NOTCH alterations promotes proliferation, migration and cellular survival via signalling through the RAS/RAF/ERK, PI3K and JAK/STAT pathways, all of which are regularly dysregulated in HNSCC. Disruption of the p53 pathway also leads to high levels of genomic instability. Green boxes show possible therapeutic agents either approved (*) or under investigation for clinical use in HNSCC. Information about the percentage of HNSCC cases showing either mutations or overexpression of the pathway as a whole shown in red boxes, where this data are available. Note, though activation of the JAK/Stat pathway is regularly seen in HNSCC, no mutations have yet been found^1–9^

HNSCCs have a high rate of genetic heterogeneity^3^, resulting in loss-of-function mutations in tumour-suppressor genes such as p53 and p16^INK4a^, and activation of oncogenes, such as the epidermal growth factor receptor (EGFR)^4^ and PIK3CA^5^. Until the recent FDA approval of immunotherapies, Cetuximab, a monoclonal antibody (mAb) targeting EGFR, has been the only targeted drug approved for both HPV-positive and -negative subtypes^6^. However, Cetuximab, and other therapies designed to target EGFR, has limited efficacy^7^ (Fig. 2).

In the absence of targeted therapies, radiotherapy remains the main treatment modality for HNSCC, and preferable for organ preservation^8^. HPV-positive patients respond better to RT, a trait not fully understood. The use of HPV as a biomarker for dose de-escalation has been considered, but the value of this approach has not been proven and concerns of under-treating these patients have taken precedence^9^.

The efficacy of RT is also substantially constrained by the presence of tumour hypoxia. Hypoxia is a biomarker of an aggressive phenotype, with higher rates of metastasis and recurrence^10^. Again, a full molecular understanding of this condition in HNSCC is lacking.

HNSCC subtypes are clinically, histologically and molecularly distinct. Yet, these diseases are treated uniformly, and with limited success. Despite research efforts, survival rates are at a deadlock. The lack of biomarkers for personalised treatment suggests an urgent need for better understanding of the intricate molecular biology of HNSCC, alongside an understanding of tumour–microenvironment interactions. This review will explore the current knowledge of HNSCC biology and highlights areas of ongoing research towards improving treatment outcomes.

## The genetic landscape in HNSCC and its clinical implications

### The TP53/RB pathway

*TP53* is a tumour-suppressor gene encoding a transcription factor^11^ with roles in maintaining genomic stability, cell cycle, DNA repair, apoptosis and senescence^12,13^. p53 is a major cellular stress sensor for DNA damage or oncogene activation^14–16^. Over 80% of HPV-negative HNSCCs have p53 mutations resulting in the loss of function^17,18^. Mutations in TP53 occur early in carcinogenesis, and are mostly associated with HPV-negative cases, due to degradation of p53 by the HPV E6 oncoprotein^19^. In both subtypes, p53 mutations are associated with poor overall survival, therapy resistance and increased rate of recurrence^17^. In response to DNA damage, p53 is regulated in a MDM2-dependent manner^20^ and activated by cell cycle checkpoint kinases CHK1 and CHK2 resulting in cell-cycle arrest and apoptosis^12^.

Amplification or overexpression of another p53 family member, *TP63*, is observed in around 80% of HNSCCs^21–23^. Of the two major isoforms produced by *TP63* (TAp63 and ΔNp63), ΔNp63 plays a major role in HNSCC pathogenesis, regulating key pathways, including cell survival and renewal, senescence suppression (by suppressing p16/INK4A) and growth factor signalling^24–26^. Recent evidence indicates that ΔNp63 influences the HNSCC metabolic microenvironment via a transcriptional programme involving HAS3 and HYAL genes, and the signalling of hyaluronic acid via CD44, activating pro-proliferative and pro-survival pathways in HNSCC^27^. Another p53 family member, p73, and its isoform TAp73 can indirectly influence growth arrest or apoptosis by regulating p53 target genes. Despite the retained DNA-binding activity of the other isoform ΔNp73, it cannot transactivate p53 reporter genes^28^. In HNSCC, TAp73 has been shown to suppress EGFR transcription and induce cell death in EGFR-overexpressing cell lines^28^. In cancers with mutant p53, proinflammatory cytokine TNF-α-induced c-REL/ΔNp63α interactions, inactivate tumour suppressor TAp73 function, promoting TNF-α resistance and survival. c-REL depletion enhanced TAp73 promoter interaction and expression of genes mediating growth arrest and apoptosis^29^. However, while amplification of *P63* is frequent in HNSCC and the consequent oncogenic function of ΔNp63 has been sufficiently understood, the extent of p73 contributions to the disease needs further investigation.

The retinoblastoma tumour suppressor (RB) regulates cell-cycle progression at the restriction point between early and late G1. As with TP53, mutations in the RB pathway are an early alteration in HNSCC carcinogenesis. The combined mutations of p53 and RB pathways result in unlimited replication potential of cancer cells^30^. In HPV-positive cancers, binding of viral protein E7 to pRb results in degradation leading to E2F release and uncontrolled cellular proliferation^30,31^.

Several strategies for p53-targeted therapies have been developed, such as adenoviral p53 gene therapy or use of small molecules to restore TP53 function or disrupt inactivation of wild-type p53. But, these have not proved effective in clinical trials^32^. Other small molecules targeting p53 for reactivation of p53 are in early-stage investigations. COTI-2 (a derivative of thiosemicarbazones) showed activity in refolding mutant p53 and restoring wild-type p53 function. A clinical trial (NCT02433626) will soon test efficacy of COTI-2 in HNSCC^33^.

### NOTCH pathway

A 2015 genomic analysis by the Cancer Genome Atlas (TCGA) showed inactivating mutations in NOTCH1-3 to be present in 17% of HPV-positive and 26% of HPV-negative HNSCCs^17^. These aberrations predominantly occur in NOTCH1 and include mis-sense mutations in functional regions, non-sense mutations resulting in truncated proteins and frameshift insertions or deletions^34^. A recent HNSCC cohort study revealed poor prognosis in NOTCH1 mutation cases, where direct downstream targets HES1 and HEY1 were overexpressed^35,36^. NOTCH1 signalling can contribute to the maintenance of cancer stem cell traits responsible for recurrence and metastasis through Wnt signalling^37^. The crosstalk between Notch and Wnt signalling has been reported in different types of cancers^38^. Loss of Notch signalling was shown to increase transcriptional activity of a β-catenin-responsive reporter construct in colon cancer stem and progenitor cells^39^. In HNSCC, concurrent NOTCH1 and FAT1 inactivating mutations drives carcinogenesis by activating β-catenin^17^. Importantly, loss of Notch signalling was found to promote tumorigenesis in HNSCC by upregulating ΔNp63, but the precise mechanisms of Notch-ΔNp63 regulation remain to be elucidated. Interestingly, Notch signalling in keratinocytes could be impaired by ΔNp63 expression, suggesting a reciprocal process between Notch and p63 in the epidermis^34^.

The data from genomic and functional studies in the lung, bladder and oesophagus support a tumour-suppressor role for Notch signalling in epithelial SCCs tumorigenesis^40^. Moreover, several in vivo models provide results consistent with sequencing data from patient samples that support a tumour-suppressive role of Notch. However, in vitro studies using HNSCC cell lines demonstrated that increased activity of Notch signalling is required for maintaining malignant behaviour^41^. It is essential to functionally validate the role of Notch signalling in HNSCC using robust in vivo models, as in vitro studies are unlikely to represent patient disease development. Collectively, whether NOTCH mutations are activating or inactivating in HNSCC remains debatable^42,43^. It may be that different types of mutations occur in different subtypes of HNSCC^35^. This therefore must be carefully considered when taking inhibitors or activators of this pathway into clinical trials^44,45^.

### PI3K/Akt/mTOR pathway

Recent molecular characterisation showed that in HNSCC, PI3K/Akt/mTOR seems to be the most frequently deregulated pathway. In HNSCC, the PI3KCA gene harbours mutations at a rate of ~16%^30^. PI3Ks are a class of enzymes vital for cellular growth, differentiation and survival, activated by RTKs, such as EGFR. Other members of the pathway include the mTOR complexes (mTORC1 and mTORC2), and Akt. mTORC2 is essential for Akt phosphorylation and activation of other signalling molecules of the PI3K pathway, including SGK1^46^.

Clinical trials have assessed the value of targeting this pathway with drugs, including rapamycin, everolimus and temsirolimus with encouraging outcomes^47^. Emergence of resistance to mTOR inhibitors has been encountered in HNSCC, though the mechanisms behind this resistance are still being investigated^48^. In one such example, a possible feedback loop between Akt and ERK/MAPK signalling by mTOR inhibition was found to act as a survival mechanism in tumour cells. Co-targeting mTOR and EGFR, thus inhibiting upstream activation of the Akt and ERK signalling pathway, has been suggested to overcome this resistance^49^.

### Epidermal growth factor receptor (EGFR) pathway

The epidermal growth factor receptor (EGFR, HER1 or ErbB1) belongs to the HER/ErbB family of receptor tyrosine kinases (RTKs), which also includes HER2-4. EGFR is overexpressed in 80–90% of HNSCC cases and correlates with poor prognosis and treatment outcomes^50^. EGFR signalling is a complicated and multidimensional network, involving many individual players and overlap with other pathways. As such, the potential for therapeutic targeting of EGFR signalling is vast, representing both a daunting challenge and tantalising opportunity for HNSCC research.

EGFR is a transmembrane receptor with tyrosine kinase activity^51^. Ligand binding triggers homo- or hetero-dimerisation with other HER members and subsequent phosphorylation of tyrosine residues, activating downstream signalling cascades. These pathways control proliferation, differentiation, survival, angiogenesis, invasion and metastasis in cancer^52^. EGFR can also translocate to the nucleus where it may function as a transcription factor. This translocation has been found to be triggered by ionising radiation, resulting in radiotherapy resistance^53^. EGFR has also been shown to interact with other receptors, such as Axl, enhancing oncogenic potential^54^.

Targeting EGFR can be achieved either by blocking the ligand-binding domain using monoclonal antibodies (mAbs), or by inhibiting the activity of the tyrosine kinase domain using small-molecule tyrosine kinase inhibitors (TKIs). Cetuximab, a chimeric monoclonal antibody with high specificity and affinity to EGFR, remains the only approved targeted therapy for HNSCC in combination with RT/CT^7,55,56^. Despite high rates of EGFR overexpression,Cetuximab has shown limited efficacy in HNSCC. This may be due to aberrations in other HER family members and their ligands, and/or activation of other downstream signalling components^57,58^.

The mechanisms behind inherent and acquired resistance toCetuximab remain unsolved^59^. Several new targeted therapies have been developed to target EGFR or its signalling partners. So far, these have shown only modest improvements in progression-free survival (PFS), and none have been approved for treatment in HNSCC (see Table 1).

**Table 1:** List of EGFR-targeted therapies in clinical trials

Drug	Target	Disease	Usage	Results of treatment	Trial
Panitumumab	EGFR	LA-HNSCC R/M-HNSCC	Combination treatment with cisplatin and fluorouracil	• Threefold decrease in disease progression • Higher number of toxicities • Increase in treatment related deaths	SPECTRUM
Zalutumumab	EGFR	R/M-HNSCC	After failure with platinum-based CT	• Extended PFS • No improvements to OS	ZALUTE
Nimotuzumub	EGFR	LA-HNSCC	In combination with C/RT	• Trial ongoing	NCT00910117 NCT00702481
Duligotuzumab (MEHD7945A)	EGFR and HER3	R/M-HNSCC	In combination with RT or in comparison to Cetuximab after failure to platinum CT	• Some evidence of radiosensitising action • Comparable PFS when compared with Cetuximab	NCT01911598 MEGHAN
Sym004	Multiple sites on EGFR	R/M-HNSCC	Patients resistant to Cetuximab	• Statistical analysis not yet posted • Downregulation of EGFR observed • Pre-clinical evidence demonstrates efficacy in delaying tumour growth and sensitising to radiation	NCT01417936
Afatinib	Pan-HER TKI	R/M-HNSCC	For patients after progression on/after platinum CT	• Improvement in PFS and disease control	NCT01538381
Erlotinib	EGFR TKI	R/M-HNSCC LA-HNSCC	In combination with Bevacizumab/in combination with Cisplatin and RT	• Modest improvements in PFS • No significant increases in PFS or CR when combined with C/RT	NCT00055913 NCT00410826

*R/M-HNSCC* recurrent/metastatic HNSCC, *LA-HNSCC* locally advanced HNSCC, *TKI* tyrosine kinase inhibitor, *RT* radiotherapy, *CT* chemotherapy, *C/RT* concurrent chemo/radiotherapy, *PFS* progression-free survival, *OS* overall survival, *CR* complete response

Small TKIs of EGFR have been ineffective in HNSCC although some early results from trials with combination therapies have shown promise. Erlotinib, for example, demonstrated modest improvements in PFS when used in combination with an anti VEGF antibody (Bevacizumab) in recurrent/metastatic (R/M)-HNSCC^60^. However, when used for locally advanced (LA)-HNSCC in combination with cisplatin and radiotherapy, the study group demonstrated no improvements in disease progression. Afatinib, a pan-HER TKI, has also been shown to have positive anti-tumour activity comparable with Cetuximab^61,62^. In a study comparing Afatinib to methotrexate, it was noted that patients who experienced improvements in disease outcomes had EGFR amplification, low HER3, no expression of p16 and high PTEN^63^, highlighting the importance of identification of biomarkers in patients who show favourable response.

### MET pathway

One proposed mechanism for resistance to EGFR-targeted therapies is upregulation or activation of other RTKs, such as c-MET (hepatocyte growth factor receptor). C-MET encodes mesenchymal–epithelial transition factor associated with increased migration, invasion and metastasis in cancer^64^. C-MET mutations are reportedly rare in HNSCC (2–13%), while gain in MET copy number and overexpression of its ligand hepatocyte growth factor (HGF) is common in HNSCCs^65^. The roles of HGF/c-MET in HNSCC invasion and metastasis have been investigated in many studies. C-MET has been found overexpressed in lymph node metastasis, and HGF has been shown to promote anoikis resistance in HNSCC, an essential step for nodal metastasis^66^. In patients, overexpression is associated with worse prognosis and lower overall survival^67^.

EGFR and c-MET share common downstream pathways, including the RAS-RAF-MAPK and PI3K-AKT-mTOR pathways. Therefore, the MET-HGF axis could represent a valuable therapeutic target in HNSCC, of particular relevance to patients with resistance to EGFR-targeted therapies^64^. Studies on dual blockade of EGFR and c-MET have reported promising anti-tumor activity of combined treatment^67^. Co-targeting both receptors has demonstrated ability to sensitise cells to EGFR-targeted therapies^68^. Capmatinib (INC-280) is a c-MET inhibitor with anti-tumour activity in mouse models. A phase I trial assessing Capmatinib safety in advanced solid tumours (NCT01324479) is completed and awaiting results^69^. Ficlatuzumab, a mAB that targets the HGF/c-MET axis, is also being tested in clinical trials for HNSCC in combination with Cetuximab in R/M-HNSCC (NCT02277197). Promising results from pre-clinical models of HNSCC showed inhibition of proliferation, migration, invasion and EMT^70^.

### JAK/STAT pathway

In both HPV-positive and HPV-negative HNSCCs, aberrant regulation of the signal transducer and activator of transcription (STAT) family has been reported. Upregulation of STAT3 and its gene targets is thought to contribute to the malignant behaviour of HNSCCs, resistance to chemo/radiotherapy and EGFR-targeted therapy^71–74^.

STAT3 signalling is considered immunosuppressive and may protect cancer cells from recognition and lysis by cytotoxic T lymphocytes, achieved by triggering production of cytokines, including IL-6, IL-10, VEGF and TGF- β1^75^. STAT3 is activated in response to upstream signals of the IL-6 cytokine receptor family, RTKs such as EGFR, VEGFR, Jenus-activated kinases (JAK) and Src family kinases (SFK)^76^. Following activation, nuclear phospho-STAT3 promotes expression of target genes including pro-survival factors, such as cyclin D1, survivin and Bcl-xL^71^.

In the context of STAT3 targeting, Ruxolitinib is an approved JAK inhibitor for myelofibrosis. A clinical trial currently in the recruitment phase aims to test efficacy of Ruxolitinib in HNSCC (NCT03153982). AZD9150, a synthetic anti-sense oligonucleotide molecule targeting STAT3 by inhibiting mRNA translation, has demonstrated anti-tumour activity in xenograft models. It is currently being tested in clinical trials of metastatic HNSCC cases as a monotherapy or combined with MED14736, an immunotherapy blocking the interaction of PD-1 and PD-L1 (NCT02499328)^77^.

### RAS/RAF/MAPK pathway

The mitogen-activated protein kinase (MAPK) pathway regulates expression of proteins involved in cell proliferation, differentiation, apoptosis, angiogenesis, invasion and metastasis^78^. It comprises four sub-pathways of which the Erk1/2 pathway has received most attention in HNSSCC. Upon binding of growth factors (such as EGF), a signalling cascade results in activated Erk1/2 that dissociates from the Ras-Raf-MEK-ERK1/2 complex and phosphorylates a number of cytoskeletal proteins, kinases and transcription factors, including NF-**κ**B, AP-1, ETS-1 and c-Myc^79^. Mutations in the MAPK pathway have been implicated in other cancers, however, in HNSCC mutations only represent 4% of cases^17,80^. MEK inhibitors, such as Trametinib, are currently approved for melanoma treatment, and are being investigated in clinical trials for HNSCC^81^.

### HPV-associated HNSCC

HPV is a risk factor associated with 22% of oropharyngeal (OPSCC) and 47% of tonsillar squamous cell carcinomas (TSCC). The incidence of HPV-positive HNSCC increased 225% from 1984 to 2004, and has now surpassed the incidence of HPV-induced cervical cancer^5^. This increase is thought to be a consequence of changes in sexual behaviour^82^. Of the 200 types of HPV viruses, HPV-16 is the most common type found in HNSCCs (90% of HPV-OPSCC), followed by HPV-18 (3%).

Clinically, HPV-positive patients often present with small tumours, but with advanced nodal metastasis. The epidemiological profile for HPV-positive patients is unique, with the majority of patients being young, white and male^9^. Crucially, HPV-positive HNSCC patients demonstrate favourable prognosis, with a 28% reduced risk of death and almost 50% reduced risk of local recurrence when compared with HPV-negative patients^83^. HPV-positive patients show improved response to radiotherapy and chemotherapy^84^. Whether this is due to the molecular pathogenesis, or related to age and better overall health of patients remains unclear.

In contrast to HPV-derived cervical cancers, in HNSCC, dysplastic lesions are rarely found before cancers are diagnosed. The HPV virus is also not commonly found in non-malignant tonsil tissue samples^85^. Evidence suggests that the tonsillar crypt epithelium on its own might not be permissive of the viral reproductive cycle. Despite this, the microenvironment is such that some 47% of infections within the tonsillar crypts can progress to cancer. Notably, only 3.9% of HPV infections in the oral cavity lead to cancer^9^.

**Fig. 3:**
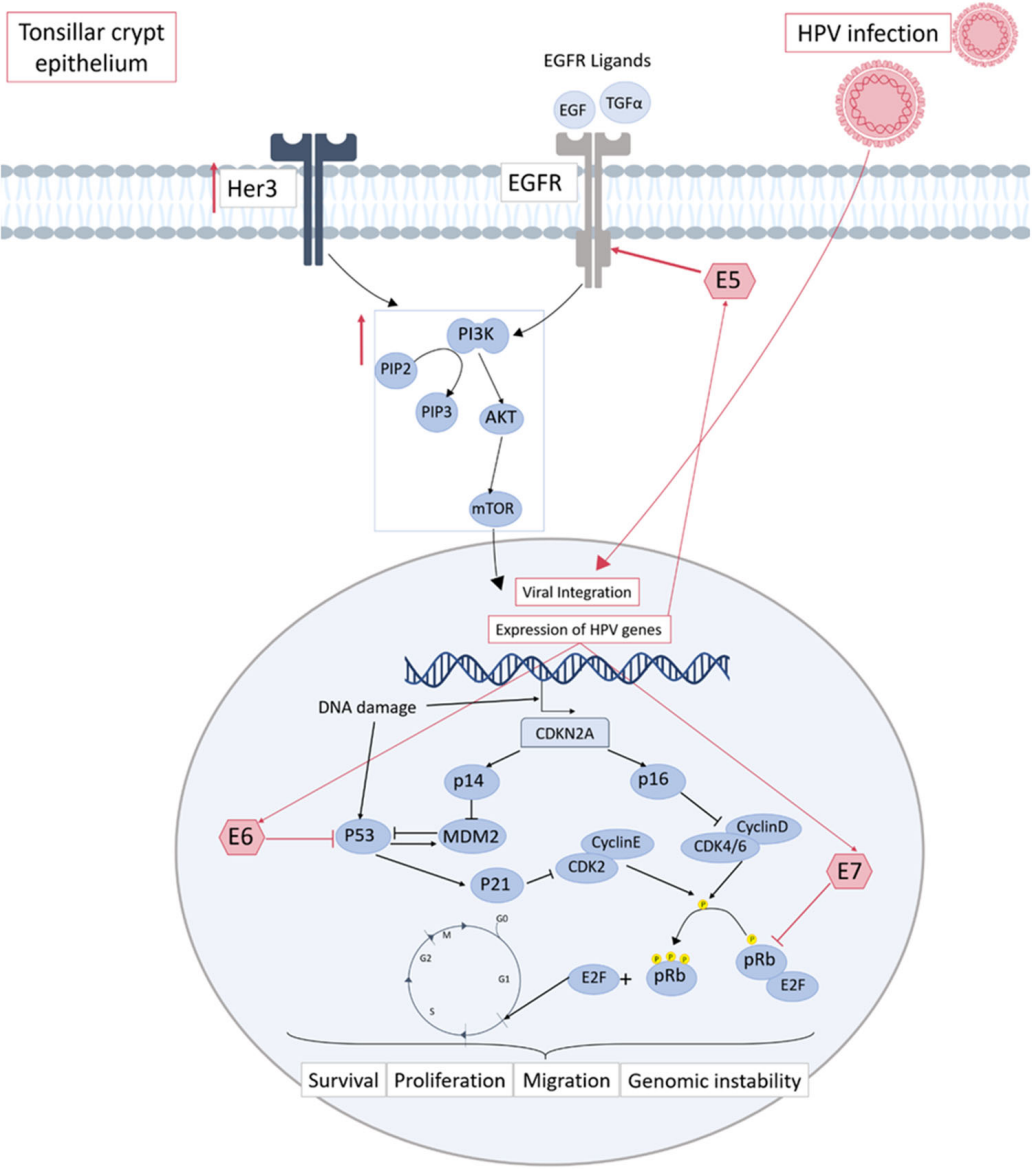
Aberrant signalling in HPV-positive HNSCC. HPV oncogenesis occurs mainly in the tonsillar crypt epithelium of the oropharynx. Upon HPV infection, viral DNA is either integrated or exists in the cells in episomes. Regardless, this allows for transcription of viral oncoproteins E5, E6 and E7. The main dysregulation attributed to carcinogenesis in HPV-positive cases stems from the inhibition of p53 by the E6 viral protein, and of Rb by E7. This leads to entry into the cell cycle via release of E2F, and inhibition of p53-mediated cell death. It also results in the accumulation of p16, which subsequently acts as a surrogate marker of HPV infection in HNSCC. Though previously considered a minor player, E5 has also been shown to activate EGFR leading to further oncogenic potential. Amongst the signalling pathways shown to be activated in HPV-HNSCC, the PI3K pathway has been found to be significantly upregulated. This may in part be due to activation by ErbB family members, including both EGFR and Her3^1,7,23–26^

Genetic alteration in HPV-derived HNSCC is primarily characterised by inactivation of p53 and pRB by viral proteins E6 and E7, respectively (Fig. 3). E5 is also implicated in carcinogenesis through activation of signalling pathways, involving EGFR, immune recognition and regulation of apoptosis^86^. PI3K mutations are observed in 30% of HPV-associated HNSCC^87^, based on current genomic data, mutation hotspots of PIK3CA vary between HPV-positive and negative tumours. Mutations predominantly occur in the helical domain of the gene in the HPV-positive subtype, whereas mutations occur throughout the gene in HPV-negative tumours^88^. These differences may have an impact on clinical outcomes to PI3K/mTOR inhibitors, and even have predictive value^48^.

TCGA data demonstrates the presence of several other genetic differences, including, loss of TRAF (a tumour-suppressor gene implicated in anti-viral immunity response) and aberrant activation of cell-cycle genes^89^. The most frequently altered genes in HPV-negative HNSCCs are often unaltered in HPV-positive HNSCCs. However, the alteration of p53, p16^INK4A^ and RB as a result E6 and E7 in HPV-positive cases is functionally similar^5^. A recent study using TCGA data, sought to further classify HNSCC tumours based on HPV status and TP53/CDKN2A mutation status. The results demonstrated that genes involved in DNA mismatch repair were upregulated in HPV-positive tumours, a pathway implicated in the cytotoxicity of chemo/radiotherapy^90^, thus providing a possible insight into the improved response of these patients to radiotherapy^91^.

Currently, there are no subtype-specific treatment regimens available^9^. Several trials have assessed de-intensification of treatment of HPV-positive cancers using various methods, including reducing total dose of radiation, RT as a monotherapy and replacing conventional chemotherapy with targeted therapies. The results of some phase III clinical trials aiming to assess efficacy of replacing platinum-based chemotherapy with Cetuximab and radiotherapy, including RTOG 1016, TROG 12.01 and the De-ESCALaTE trial^92–95^, are published. These trials report that EGFR inhibition by Cetuximab conferred a reduction in overall survival and tumour control when compared with cisplatin. The authors of the De-ESCALaTE trial also recommend caution when considering reducing the dose of radiotherapy for HPV-positive patients. Additional efforts have been made towards the development of therapeutic HPV vaccines^96^.

### Database analysis of head and neck squamous cell carcinomas

Following the omics revolution, the amount of data generated with the aim of uncovering biomarkers, network motifs and susceptibility markers has soared. The development of databases to the structure and sharing of these data has been critical. See Sepiashvili et al. for detailed biomarker data with respect to HNSCC^97^.

Both genomic and proteomic data can be accessed, with databases such as the GEO omnibus^98^ and Array Express^99^ containing microarray data, DNA- and RNA-sequencing, and DNA methylation studies (Table 2) and The Human Protein Atlas containing results from tumour tissue staining^100,101^. These databases have their own merits and pitfalls. Whereas GEO and Array Express comprehensively gather all species and cell types, the Cancer Genome Atlas (TCGA) specifically focuses on patient-derived samples and contains information about genomic changes in different cancer types. For head and neck, this includes 44 healthy and 504 cancer samples^102^. TCGA comprises mRNA, miRNA and protein expression data as well as DNA methylation status. The Leipzig Health Atlas contains gene expression profiling and targeted sequencing of 50 genes of 290 consecutively recruited HNSCC patients^103^. For metabolic alterations observed in HNSCC a variety of medium and small-scale data sets have been generated as summarised by Shin et al.^104^. Furthermore, a genomic information portal for HNSCC cell systems has been set up, detailing clinical and genomic information of 44 cell lines^105^.

**Table 2:** Some examples of publicly available data from studies involving genomic profiling of HNSCC samples

Study by	Type	Array identifier
Farah et al.^126^	microRNA profiling by array	E-MTAB-6470
Hess et al.^127^	microRNA profiling by array	E-MTAB-5198
Bossi et al.^128^	Transcription profiling by array	E-GEOD-65021
Wood et al.^129^	RNA-seq of coding RNA	E-GEOD-72536
Selvi et al.^130^	Transcription profiling by array	E-GEOD-75029
Wichmann et al., Leipzig Head and Neck Group, 2015^120^	Transcription profiling by array	https://www.health-atlas.de/en/

The Danish Head and Neck Cancer group (DAHANCA) established in 1976, was set up with the aim of generating guidelines for the treatment of HNSCCs based on patient cases. To date, they have compiled data from more than 33,000 patients containing variables incorporating symptoms, aetiological factors, diagnostic evaluation and involvement in clinical trials^106^. This database is a valuable resource for longitudinal studies, which have clear advantages over single case studies. However, the accessibility of DAHANCA to the scientific community is limited by the use of the Danish language in all reports. The National Cancer Database (NCDB), based in the USA, collects information submitted from hospitals detailing the state of cancer care throughout the country. Between 1990 and 2004, information of around 800,000 head and neck tumours were collected^107^.

In contrast to these large studies, some databases have attempted to group all decentralised information into a single source. One such database for head and neck cancer is HNdb, which was established with the aim to combine genomics, transcriptomics and proteomics data, literature citations, and cross-references from external databases. For this purpose, Henrique et al. established a text mining procedure to identify genes in literature related to HNSCC. Their database provides user-friendly access to information on HNSCC-related genes and their different biological data resources^108^. Overall, the large amounts of data amassed so far offer a great foundation for the identification of possible biomarkers, to begin working towards personalised medicine for HNSCC.

### The microenvironmental landscape

#### Hypoxia in HNSCC

Hypoxia is a well-established cause of poor response to treatment, metastasis and recurrence. It develops as a result of progressive tumour growth leading to impairment of oxygen supply within tumour tissues. This results in the development of areas starved of oxygen^30^. Within these regions, central areas (experiencing oxygen levels < 0.1% O_2_) will likely become necrotic. The cells within regions of acute hypoxia (< 2% O_2_) that survive these extreme conditions adapt a programme of gene expression leading to treatment resistance and metastasis^109–111^.

The hypoxic response is primarily mediated by the heterodimeric hypoxia-inducible factors (HIF-1-3). Under normal oxygen conditions, the HIFα subunits undergo rapid degradation by the E3-Ubiquitin Ligase Von-Hippel-Lindau protein (VHL). Under hypoxic conditions, HIFα is stabilised and binds to HIFβ in the nucleus, where the activated HIF complex binds to hypoxia response elements (HREs), allowing for adaption of the tumour. HREs are present in genes responsible for altering metabolism (CA9, GLUT1), EMT (Vimentin), extracellular matrix remodelling (LOX, MMPs), angiogenesis (VEGF), immune modulation and inflammation (TNFα, IL1β)^112–116^.

The state of hypoxia is not clear-cut, with several examples of stabilisation of HIFs in normoxic conditions. mTOR has been shown to induce expression of HIF-1α in tumour areas that are not significantly hypoxic, and has been attributed to various oncogenic mechanisms, such as inactive p53 mutations, excessive accumulation of oxygen radicals, RAS mutations, inactivation of PTEN and ineffective degradation of HIF-1α by mutations in VHL^112^. Gain-of-function mutations in p53 have shown a synergism with HIF signalling. A recent study in hypoxic NSCLC found p53 mutations that regulate a selective gene signature including extracellular matrix (ECM) components, such as type VIIa1 collagen and laminin-γ2. This study illustrated the impact of p53 mutants on the microenvironment in co-operation with HIF-1 to promote cancer progression^117,118^. By considering the high frequency of p53 mutations in HPV-negative HNSCC and the hypoxia observed in these tumours, the co-operation between p53 mutants and HIF-1 signalling might represent a meaningful avenue for clinical investigation.

Hypoxic tumours represent a distinct subtype of HNSCCs with poor prognosis and treatment resistance. Attempts have been made to make clinical use of this information. Hypoxic modulation has been suggested to revert the changes that occur in low oxygen and restore sensitivity to treatment. Hyperbaric oxygen therapy is one such method which aims to improve tumour oxygenation^119,120^. Hypoxia-activated pro-drugs including Tirapazamine and Nimorazole are thought to act as oxygen-mimetic radiosensitizers. The latter is now included in the standard of care for Danish HNSCC patients, following guidelines from DAHANCA^110,121^. A trial using Nimorazole alongside RT for patients with R/M-HNSCC is currently recruiting in the UK (NIMRAD)^122^.

Targeting hypoxia therapeutically to overcome treatment resistance needs accurate and reliable detection methods. Several approaches including needle electrodes, endogenous or exogenous hypoxia tissue markers and hypoxia imaging have been tested, but have limitations. Needle electrodes are an invasive method limited to accessible tumours, albeit with demonstrable prognostic value in HNSCC tumours^123^. To overcome these limitations, endogenous hypoxia biomarkers have been identified in patient tissues and serum, the most commonly used being GLUT-1, CA9, VEGF and serological biomarker OPN^124^. Several hypoxia-specific gene signatures (HGS) have been developed^125–127^, with evidence of HGS having prognostic and predictive values. Notably, a 15-gene HGS from human HNSCC xenografts has been identified and further validated in a cohort of 302 HNSCC patients with successful discrimination between low and high hypoxic tumours^127^.

Positron emission tomography (PET)/computer tomography (CT) using hypoxia-specific radiotracers, combined with assessment of endogenous or exogenous hypoxia markers could provide a reliable clinical method for both detection and measurement of tumour hypoxia during treatment^126,128^. In HNSCC, PET imaging using the hypoxia radiotracer ^18^F-MISO has been investigated, demonstrating successful identification of HNSCC patients who benefited from addition of Tirapazamine with prediction of recurrence after radiotherapy^129^. Likewise, a recent study by Suh et al. combined hypoxia imaging with PET/CT using ^64^Cu-ATSM and gene expression of patient tumours to validate a HGS, linked to prognosis^126^.

#### The immune-microenvironment in HNSCC

Immune evasion is considered a key hallmark of cancer, generating an environment permissive of survival and progression. HNSCCs are immune-suppressive, with the ability to avoid recognition and clearance by immune cells. This evasion of immunosurveillance is achieved by alterations, including loss or downregulation of human leucocyte antigens (HLAs) expression, impaired recognition of cancer cells by T cells and activation of MAPK, STAT3 and β-catenin/Wnt signalling pathways^130^. The HNSCC tumour microenvironment has also been shown to have impaired function of tumour-infiltrating lymphocytes (TILs)^131^.

Both HPV-negative and HPV-positive tumours show high levels CD8 + cytotoxic T cells and activated NK cells^132^. However, these tumours still evade their cytotoxic mechanisms. For clinical purposes, research is ongoing to investigate efficacy of enhancing immune cell targeting of tumours. A potential method for this could be to exploit the presence of tumour-associated neoantigens expressed on cancer cells resulting from genetic reconfigurations^133^.

Adoptive immunotherapy, which involves administration of genetically modified T cells targeting specific antigens expressed on the surface of tumour cells, is a recent revolutionary advance in cancer immunotherapy. The synthetic chimeric antigen receptor (CAR)-T cell therapy has shown remarkable success in haematological cancers^134^. An FDA-approved CD19-targeting CAR-T cell therapy has demonstrated success in leukaemias and lymphomas, encouraging the development of similar therapies for solid tumours^135^. However, these therapies face multiple challenges such as a need to identify specific tumour-associated antigens that are overexpressed in tumours, but not in normal tissues^136^. A recent ongoing trial developed a pan-ErbB-targeted CAR-T cell therapy for HNSCC (NCT01818323)^137^.

The momentous discovery that certain proteins act as “immune checkpoints” by regulating the T-cell response has spurred efforts to develop treatments to reverse this effect and restore anti-tumour immune responses^138,139^. In normal circumstances proteins including programmed death protein (PD-1), its ligand (PD-L1) and cytotoxic T-lymphocyte-associated protein 4 (CTLA-4), function to prevent autoimmunity^140,141^. PD-L1 has been detected in most human cancers and leads to anergy and apoptosis of activated T cells and subsequent immune evasion of the tumour cells^142–145^. These proteins are often overexpressed and co-opt this protective mechanism to inhibit immune activation. Immune checkpoint blockade therapies have shown remarkable clinical success for some cancers, including metastatic melanoma^140,146,147^. In 2016, anti-PD1 mAb Nivolumab was FDA-approved for recurrent/metastatic HNSCC patients with progressive disease or failure of platinum-based therapy. The approval followed the promising results of the CheckMate 14 trial, demonstrating a statistically significant improvement in OS and quality of life in patients treated with nivolumab^148^. Pembrolizumab, another anti-PD1 mAb, was in the same year approved in R/M-HNSCC treatment after the positive results of the KEYNOTE-12 trial^149^. Studies to approve these drugs as mono or combination therapies for different cancer types is ongoing.

#### Cancer-associated fibroblasts

Cancer-associated fibroblasts (CAFs) are fibroblast-like cells associated with the tumour that develop an activated phenotype, expressing markers such as α-smooth muscle actin (α-SMA) and fibroblast activation protein (FAP)^150^. These fibroblasts, alongside infiltrating immune cells, can resemble the architecture of a wounded tissue. However, unlike wound fibroblasts, CAFs are often resistant to cell death mechanisms and remain activated^151–153^. Communication, through direct contact and secretion of signalling molecules between CAFs and tumour cells is vital for their function^154^. With perpetual activation of CAFs, the tumour can benefit from CAF secretion of proteins for ECM remodelling^155^, growth factors^152^, cytokines, chemokines and recruitment of immune-suppressive cells^156^. This environment supports tumour growth, migration, invasion, angiogenesis, colonisation of distant tissues by way of the CAF-supported “pre-metastatic niche” and evasion of the immune system^157–161^. An additional danger engendered by this stroma–tumour communication has been the reported changes in responsiveness to cancer therapy^161–163^. This is particularly relevant in HNSCC, as CAFs have been shown to regulate response to Cetuximab^164^ and radiotherapy^165,166^. Research has shown that these treatments in turn impact CAF activation^167,168^.

A number of potential targeting strategies exist to block CAF-mediated tumour support, including inhibition of CAF cell-surface proteins (such as the anti-FAP antibody Sibrotuzumab^169^), blocking CAF activation or by targeting CAF-tumour signalling^159^. Tumour cells are also thought to contribute to activation of CAFs via a transformation process involving exosomes containing nucleic acids like microRNAs^170,171^, chemokines and cytokines like TGFβ, CXCL12 (CXC-Chemokine ligand 12 also known as SDF1) and IL-6, as well as local stimuli like hypoxia and oxidative stress^154,156,172^. Though research within this field with respect to HNSCC is somewhat underdeveloped, many of these factors have independently been implicated in its oncogenesis^173–177^. Recent work by Hersi et al. showed that low expression of the tumour-suppressive miR-9, which targets the CXCL12 receptor CXCR4, was linked to aggressive behaviour of HNSCC cells, counteracted by targeting of this interaction with Plerixafor (a CXCR4 inhibitor)^178^. Likewise, IL-6, its receptor and downstream JAK/STAT signalling have all been strongly implicated in HNSCC prognosis^71,179^. A recent study showed that IL-6 was responsible for both promoting oncogenesis when secreted from CAFs and CAF activation when secreted from tumour cells^180^.

Importantly, targeting some of these mechanisms, such as the CXCL12-CXCR4 and IL-6 pathways, could result in inhibition of both the tumour stroma and the tumour itself. One challenge associated with targeting CAFs in the HNSCC microenvironment relates to their heterogeneity. Their biology is complex, and CAFs likely originate from a number of different cell types^181^. In practice, this non-uniform pool of cells has been difficult to characterise completely. One benefit of CAFs is that they are genetically stable, unlike tumour cells, which may make long-term treatment plans more viable^159^. As with much of what has been reviewed herein, holistic approaches involving combinations of immune therapy, conventional therapy and CAF-targeted therapy may prove most useful for future treatment.

#### The oral microbiome

Changes in the oral microbiome have been proposed to contribute to oncogenesis in the 7–15% of oral cancer cases, which cannot be explained by known risk factors^182–184^. The microbiome denotes the collective genome of complex communities of bacteria, archaea, viruses, fungi and protists, each with crucial roles to play in stabilising microbial diversity^185,186^. The clinical relevance of the oral microbiome lies in the statistical association between dysbiosis (often a result of poor oral health) and the prevalence of many types of cancer^187–189^. Within the multifarious environment of the human mouth, with its mucosal surfaces and deep-tissue crevices, both healthy and malignant sites contain distinct microbial populations^190,191^.

Several bacterial species have been associated with oral cancer. A potential biomarker signature has been suggested for oral cancer and consists of the three bacterial species *Capnocytophaga gingivalis*, *Prevotella melaninogenica* and *Streptococcus mitis*. These bacteria are found in 80% of OSCC cases, and the signature has demonstrated a diagnostic sensitivity of 80% and a specificity of 82%^192^. Another three-fungal signature of *Rhodotorula, Geotrichum* and *Pneumocystis*, as well as the microsporidia *Phialophora* and *Cladophialophora* are specifically seen in OCSCC^183^.

Risk factors for HNSCCs including tobacco and alcohol consumption and HPV infection have been shown to affect the oral microbiome^193–196^. A shift towards different genera of bacteria has been associated with exposure to these risk factors. Oral microbes have also been shown to contribute to acetaldehyde (the carcinogenic metabollite of ethanol) production, which can induce mutagenesis, and hyperproliferation of the epithelium^197^. Bacterial strains show significant differences in their ability to produce acetaldehyde, for instance, *S. mitis* produces high amounts of acetaldehyde and has significant alcohol dehydrogenase activity^198^. Concurrently, elevated levels of *S. mitis* have been detected in OSCC^192^. Acetaldehyde and malondialdehyde production from *Streptococcus* species such as *Streptococcus gordonii V2016* significantly increased bacterial attachment to keratinocytes, facilitated HPV infection by enhanced expression of furin and resulted in malignant transformation of infected keratinocytes^199^. Oral *Streptococcus* species and HPV seem to co-operate in order to infect and transform oral keratinocytes after exposure to alcohol. Cleavage of the minor capsid protein L2 by furin is required for HPV infection^200,201^. Since only a subset of bacteria contains enzymes with furin-like activity, this might contribute to HPV tissue tropism^202^.

The carcinogenic potential of several bacteria has been demonstrated in vitro and in animal models^184^. Chronic infection *of P. gingivalis* or *F. nucleatum* have been shown to augment the IL-6-STAT3 inflammatory cascade and promote HNSCC development^203^. Interactions between periodontopathogenic bacteria and single-nucleotide polymorphisms (SNPs) of Toll-like receptors (TLRs) such as TLR2 and TLR4 have also been shown to influence OSCC risk^204–206^. Virulent *P. gingivalis* strains can induce expression of PD-L1 and PD-L2 receptors in squamous carcinoma cells, mediated by the membrane fraction of *P. gingivalis*^207,208^.

Particularly relevant for HNSCC and its treatment options, some microbiota such as *C. albicans* and *E. faecalis* can activate EGFR signalling^209–211^. In addition, *E. faecalis* can drive tumorigenesis by hydrogen peroxide-mediated DNA damage, chromosomal instability and mutagenesis^211^. The compositional and functional variations of oral microbiota have been associated with the mutational changes in oral cancer. Yang et al. investigated the oral microbiome composition of OSCC patients in association with their mutational profile and identified three patient clusters, which varied in their bacterial species richness and their relative abundance of Firmicutes and Bacteroidetes^212^.

The clinical relevance of the microbiome is important as treatments such as radiotherapy cause alterations in healthy oral microflora, partially due to treatment-induced xerostomia^213–215^. Ultimately these alterations can result in exacerbation of mucositis and systemic infections. Patients who have good oral care during cancer therapy have better outcomes^213^. Different treatments in oral cancer patients have distinct effects on microflora^216^. Opportunistic pathogens such as staphylococci, enteric rods and *Candida sp*. tend to increase in prevalence after radiotherapy (IMRT) with or without chemotherapy^217^. *C. albicans* was found in one or more sites in 54% of patients who received radiotherapy in comparison with 15% of controls^188^.

A new avenue for personalised treatment could involve targeting the microbiome for therapeutic purposes with microbial supplements, such as synbiotics (probiotics and prebiotics), diet or microbial suppression strategies using antibiotics^218–223^. Probiotics have the potential to protect against cancer development in animal models, and some probiotic strains diminish the incidence of postoperative inflammation in cancer patients^224–229^. Importantly, most commercial probiotic products are generally safe and can improve the health of the host by modulating the intestinal microbiota and immune response^230^. Therefore, probiotic strains might be useful adjuvants for cancer prevention and/or treatment^231^. *Lactobacillus brevis* CD2 lozenges have been shown to reduce the severity and incidence of radio/chemotherapy-induced mucositis in HNSCC patients, thereby increasing the rate of anticancer treatment completion^232,233^.

In brief, the emerging fields of microbiomics and metagenomics will help to identify the presence of HNSCC-specific microbes and help us understand^234^ the development of accurate and cost-effective diagnostic and therapeutic strategies.

## Conclusion

The heterogeneous nature of HNSCCs has hindered the identification of specific targets for the development of targeted therapies. Over the past 30 years we have developed a better understanding of the genomic, proteomic, microbiomic and metabolomic alterations in HNSCCs. This knowledge is helping to move us closer to personalised therapy, where each subtype can be treated as a separate disease. The significant problems associated with high toxicities as well as resistance to current treatments, and low quality of life for patients, make these efforts particularly crucial. Since our last clinical update in 2014^4^, our understanding of these diseases has broadened to not only consider the endogenous alterations as key contributors to oncogenesis but also to consider the microenvironmental factors that build an environment permissive of these oncogenic mechanisms. How the integration of microenvironmental factors and genetic backgrounds reshapes the HNSCC epigenome is largely unclear and represents one of the new priorities in the field. It is likely that interdisciplinary approaches seeking to link the genetic and microenvironmental biology of HNSCC will allow us to better treat HNSCC in the future.
